# Supplementary material for: Realization of all-optical vortex switching in exciton-polariton condensates
Source: Nat Commun. 2020 Feb 14;11:897. doi: 10.1038/s41467-020-14702-5 (PMC7021691; doi:10.1038/s41467-020-14702-5)
Supplement: Supplementary file 1 — Supplementary Information [file 41467_2020_14702_MOESM1_ESM.pdf]

Supplementary Information: Realization of all-optical vortex switching in exciton-polariton condensates

Xuekai Ma,[1,*] Bernd Berger,[2] Marc Aßmann,[2] Rodislav Driben,[1] Torsten Meier,[1] Christian Schneider,[3] Sven Höfling,[3,4] and Stefan Schumacher[1,5]

[1] *Department of Physics and Center for Optoelectronics and Photonics Paderborn (CeOPP), Universität Paderborn, Warburger Strasse 100, 33098 Paderborn, Germany*
[2] *Experimentelle Physik 2, Technische Universität Dortmund, 44227 Dortmund, Germany*
[3] *Technische Physik, Physikalisches Institut and Würzburg-Dresden Cluster of Excellence ct.qmat, Universität Würzburg, Am Hubland, 97074, Würzburg, Germany*
[4] *SUPA, School of Physics and Astronomy, University of St. Andrews, St. Andrews KY16 9SS, United Kingdom*
[5] *College of Optical Sciences, University of Arizona, Tucson, AZ 85721, USA*

SUPPLEMENTARY NOTE 1 - CORRELATION MEASUREMENTS OF THE $m = +1$ AND $m = -1$ OAM STATES

The simultaneous appearance of the two m=+1 and m=-1 modes raises the question if these modes coexist or if a single OAM mode forms in each individual excitation cycle. Both scenarios may result in the same time-averaged image. To investigate this question, we modify the experimental setup such that vortices are spontaneously created using only the now ring-shaped pulsed excitation laser. Supplementary Figure 1(a) shows the real space image of the condensate emission and the position of the pulsed excitation laser is indicated. As one can see from the OAM-resolved image in Supplementary Figure 1(b) contributions with opposite topological charges $m = +1$ and $m = -1$ form at the same time. We now perform correlation measurements where individual photon detection events for each single shot are captured and the photon correlation is calculated [1, 2]. Here we calculate the cross-correlation between two bins corresponding to the m=+1 and m=-1 modes as follows:

$$C_{-1,1} = \frac{< I_{-1} \cdot I_1 >}{< I_{-1} >< I_1 >} \, . \tag{1}$$

Here I is the photon count in the denoted bins. The averaging is done by summation of the I_{-1}, I_1 and $I_{-1} \cdot I_1$ terms for all individual shots and division by the total number of shots captured. We obtain a value of $C_{-1,1} = 1.583 \pm 0.102$. This is clear evidence for coexistence of the $m = +1$ and $m = -1$ states. If the photon events of the OAM modes in both bins were statistically independent from each other, the term $< I_{-1} \cdot I_1 >$ would separate into $< I_{-1} >< I_1 >$, resulting in $C_{-1,1} = 1$, corresponding to independent existence of the OAM modes. If anticorrelations between the OAM modes exists, so that the two modes would tend to suppress each other, the cross-correlation is expected to be $C_{-1,1} < 1$. However, when fluctuations of the OAM modes are correlated and the photon events in both bins tend to appear simultaneously, the cross correlation is $C_{-1,1} > 1$. In this case the OAM modes certainly coexist and their cross-correlation most likely originates from shot-to-shot fluctuations of the reservoir, which feeds both modes.

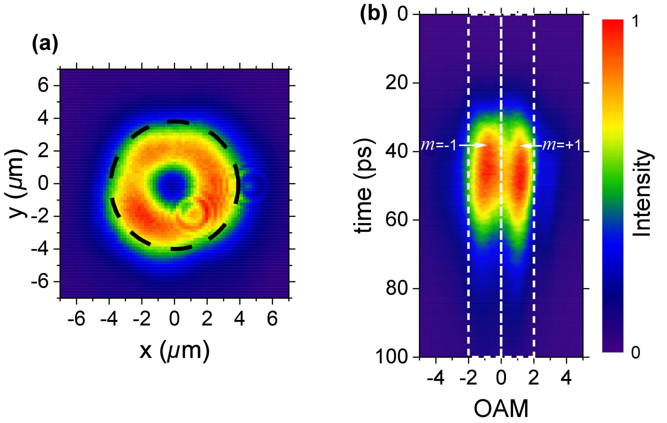

Supplementary Figure 1: **Correlation measurements of $m = +1$ and $m = -1$ OAM states** (a) Real space image of vortices forming under annular pulsed excitation with 120 fs pulses. The black circle represents the excitation ring with around 8 μm diameter. (b) The time-resolved OAM measurement shows the formation of m=+1 and m=-1 contributions at the same time. Since this image is averaged over millions of individual excitation cycles, it does not yield information on whether both OAM states coexist or are mutually exclusive. To gain insight into this question, we calculate the cross-correlation between the m=+1 and m=-1 states as indicated by the two white boxes.

SUPPLEMENTARY NOTE 2 - VORTICES MEASURED AT DIFFERENT SAMPLE POSITIONS

The switching dynamics are influenced by the local landscape and strength of sample disorder. For certain disorder configurations and weak disorder, the structure of the condensate mode can be dominated by the pump profile. As an example, in Figs. 5(a) of the main

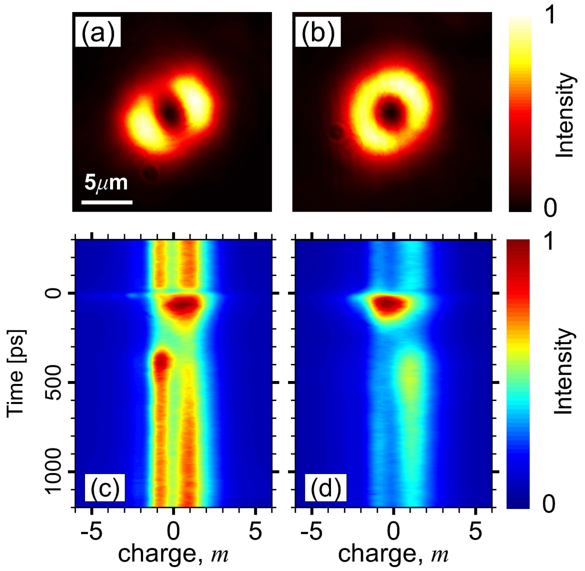

Supplementary Figure 2: **Vortices at different sample positions.** (a,b) Real-space representation of normalized measured condensate photoluminescence at different sample positions. (c,d) Time traces of photoluminescence, corresponding to (a,b) respectively, emitted from the polariton condensate, optically resolved into OAM modes. The control pulses are applied at $t = 0$ ps in (c) and (d).

text we find that the emission profile is oriented almost along the intensity valley of the imperfect pump profile in Fig. 5(b) of the main text. For this case the measurements show that the switched vortex only very slowly (after several nanoseconds) and smoothly returns to the preferred state, that is the state before switching. To better illustrate the influence of different local disorder configurations, in Supplementary Figure 2 we show results measured at different positions (here we deliberately choose bad sample positions for the switching dynamics) on the sample with the same ring-shaped excitation profile shown in Fig. 5(b) of the main text. Condensate emission profiles before application of the switching pulse are shown in Supplementary Figures 2(a) and (b). Corresponding switching dynamics are shown in Supplementary Figures 2(c) and (d). For different sample positions we observe different emission profiles from the condensate. However, as the data in Supplementary Figures 2(c) and (d) clearly illustrate, a more vortex-like emission profile does not necessarily lead to a better switching performance or switching contrast. Compared to the results shown in the main text, the switched vortex in Supplementary Figure 2(c) more rapidly reverts back to its initial state at about $t \sim 700$ ps. In Supplementary Figure 2(b) the influence of the sample disorder increases such that even with the imperfect ring-shaped excitation profile an almost ring-shaped emission profile is observed from the condensate. However, as shown in Supplementary Figure 2(d) in this case the switched vortex returns even more quickly to the initial state. These results demonstrate that the fundamental vortex switching is quite robust and works at different sample positions but it also illustrates that the local disorder landscape influences both the initial vortex state formed, stability and persistence of switching, and the switching dynamics.

SUPPLEMENTARY NOTE 3 - QUANTIFICATION OF THE COEXISTING $m = +1$ AND $m = -1$ OAM MODES

So far we have discussed our results with a focus on the optical raw data measured in the experiments. In this section, we would like to quantify further the different orbital angular momentum contributions $m = +1$ and $m = -1$ in the condensate emission. To this end, one needs to convert the continuous distribution measured in the experiment into a discrete distribution describing the relative weights of the integer OAM states. While a system may also show non-integer vorticity in principle, this cannot be a stable eigenstate of the system. Further, non-integer vortices would emit light fields with fractional OAM, which are known to be unstable upon propagation and would dissolve into integer OAM components for the propagation distances encountered in our experiment. Accordingly, it is sufficient to consider only vortices of integer topological charge. In principle, this can be done via binning the sorted intensity. However, it is known that there is crosstalk between different bins due to partial overlap of the sorted OAM states [3]. In order to arrive at a reasonable estimate of the relative OAM components, this effect needs to be taken into account.

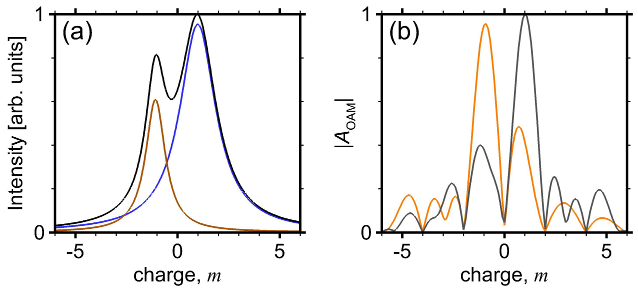

Supplementary Figure 3: **Ratio of the coexisting $m = \pm 1$ OAM states.** (a) The black line is a two-mode fit of the OAM resolved photoluminescence measured at $t = -200$ ps in Fig. 3(a) of the main text. The brown line and blue line are the individual contributions of the two modes of $m = -1$ and $m = +1$, respectively. (b) Numerically resolved OAM amplitude, $|A_{\mathrm{OAM}}|$, for the vortices shown in Fig. 5(c) (gray line) and Fig. 5(e) (orange line) of the main text.

The peaks of the individual modes take approximately Lorentzian shape. As a second effect, the modes do not necessarily have the same width, but may be smeared out. In OAM-sorting experiments, the OAM of the signal is always measured with respect to the center of the OAM transformation pattern. If the middle of the vortex and the center of the transformation pattern do not coincide exactly, the OAM-sorted signal will acquire a small offset. Unless the vortex diameter matches the confining potential exactly, it is expected that the vortex centers may move around within the confining potential and do not always coincide with the center of the potential which is imaged onto the center of the transformation pattern. Accordingly, a broadening of the peaks is expected, which results in additional crosstalk and correspondingly in a Voigt-like peak profile. As the healing length and vortex diameter scale with the inverse square root of the polariton population, modes of higher intensity are expected to show a larger broadening of the OAM-sorted spectra due to the smaller vortex sizes. A two-mode fit to the OAM-resolved emission at t=-200 ps in Fig. 3(a) of the main text is shown in Supplementary Figure 3(a). As expected, the stronger $m = +1$ mode is broader compared to the weaker $m = -1$ mode. The relative intensity weight of the $m = +1$ and $m = -1$ modes deduced from the peak areas amounts to approximately 2.89:1. This roughly corresponds to the contrast in the vortex switching operation demonstrated in the present work. However, we would like to note that in the present work neither the optical setup nor the sample were optimized to achieve optimum switching performance or contrast. In our numerical simulations, the amplitude of signals in specific OAM channels can be extracted from the condensate field Ψ at a fixed point in time as

$$A_{\mathrm{OAM}}(m) = \int \Psi(\mathbf{r})\mathbf{e}^{-im\phi}\mathbf{dr}\,. \qquad (2)$$

Here ϕ is the polar angle and the origin of the coordinate system is located in the center of the ring-shaped excitation beam. For the condensate vortex states including the influence of sample disorder shown in Fig. 5(c,e) of the main text, $|A_{\mathrm{OAM}}|$ is shown in Supplementary Figure 3(b). Including the influence of sample disorder and resulting broken rotational symmetry of the system, also the numerical results clearly show the coexistence of OAM modes with $m = +1$ and $m = -1$ before and after vortex switching, with the clear dominance of one of these modes in either case. The contrast ratio obtained in the numerical simulations is higher than that in the measurements mostly as numerical OAM sorting does not result in a significant background signal at other OAM values.

SUPPLEMENTARY NOTE 4 - NUMERICAL RESULTS FOR VORTEX SWITCHING WITH HIGHER TOPOLOGICAL CHARGES

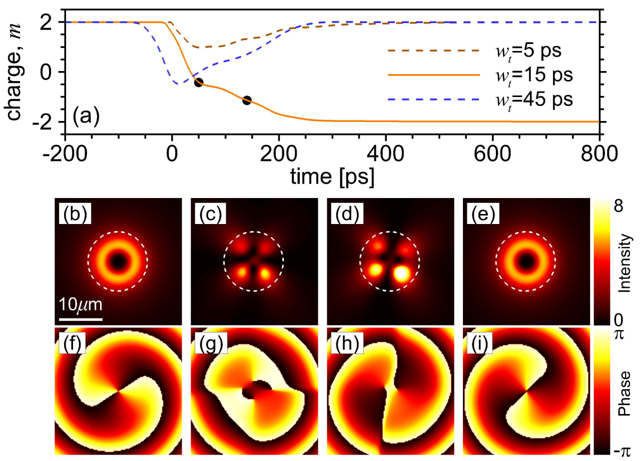

Supplementary Figure 4: **Numerical switching of vortices with higher topological charge $m = \pm 2$.** (a) Time evolution of the total topological charge of the polariton condensate for different duration of the control pulse, launched at $t = 0$ ps, with $w_t = 5$ ps, $w_t = 15$ ps, and $w_t = 45$ ps, respectively, for fixed intensity with $a = 2$. (b-e) Snapshots of density and (f-i) phase of polariton condensates at different times for the solid orange line in (a). The ring pump is indicated by the white dashed circle. (b) The initial vortex state with $m = 2$ at $t = -200$ ps. (e) The final vortex state with $m = -2$ at $t = 2000$ ps. We note that after 800 ps no significant dynamical changes are observed anymore. (c,d) The intermediate states at $t = 50$ and 140 ps, corresponding to the black points in (a), respectively.

Vortices carrying higher topological charges can also be switched between clockwise and counter-clockwise rotations by applying an additional off-resonant pulse, indicating that this method is quite generally applicable to off-resonantly excited polariton vortices. Supplementary Figure 4 shows an example of the switching dynamics of a higher order vortex from the topological charge $m = 2$ to $m = -2$. The higher order vortex is created by a CW pump with a larger radius, that is $w = 7$ μm. When it reaches the steady state with $m = 2$ [see Supplementary Figure 4(a) at $t < 0$ ps and Supplementary Figure 4(b,f)], a narrow, $w_p = 2$ μm, Gaussian pulse is applied at $t = 0$ ps at the same spatial position as in Fig. 2 of the main text. Again, we find that when the pulse is too short, $w_t = 5$ ps, the perturbation is not strong enough to stop the rotation of the vortex, so that the topological charge reduces a little and then return to the previous steady state as shown by the dashed brown line in Supplementary Figure 4(a). For a longer pulse

with $w_t = 15$ ps the topological charge first takes slightly negative values and an intermediate quadrupole mode is formed when the rotation is stopped as shown in Supplementary Figure 4(c,g). After the pulse induced perturbation has sufficiently decayed, the system continues to rotate at negative topological charge [Supplementary Figure 4(d,h)] until a persistent clockwise rotation of the condensate with $m = -2$ as a stable stationary state is reached. As mentioned in Fig. 4 of the main text for vortices with $m = \pm 1$, the switching dynamics is related to the oscillation times of the perturbed system. Therefore, also for $m = \pm 2$ an even longer pulse with $w_t = 45$ ps switches the vortex back to the $m = 2$ state [blue dashed line in Supplementary Figure 4(a)].

SUPPLEMENTARY REFERENCES

* xuekai.ma@gmail.com

[1] Aßmann, M. *et al.* Measuring the dynamics of second-order photon correlation functions inside a pulse with picosecond time resolution. *Opt. Express* **18**, 20229–20241 (2010).

[2] Schmutzler, J. *et al.* Influence of interactions with non-condensed particles on the coherence of a one-dimensional polariton condensate. *Phys. Rev. B* **89**, 115119 (2014).

[3] Berkhout, G. C., Lavery, M. P., Courtial, J., Beijersbergen, M. W. & Padgett, M. J. Efficient sorting of orbital angular momentum states of light. *Phys. Rev. Lett.* **105**, 153601 (2010).
